# Smartphone Applications for Educating and Helping Non-motivating Patients Adhere to Medication That Treats Mental Health Conditions: Aims and Functioning

**DOI:** 10.3389/fpsyg.2017.01769

**Published:** 2017-10-11

**Authors:** Angelos P. Kassianos, Giorgos Georgiou, Electra P. Papaconstantinou, Angeliki Detzortzi, Rob Horne

**Affiliations:** ^1^Department of Applied Health Research, University College London, London, United Kingdom; ^2^Department of Psychology, University of Cyprus, Nicosia, Cyprus; ^3^Cyprus Institute of Neurology and Genetics, Nicosia, Cyprus; ^4^Private Practice, Athens, Greece; ^5^School of Pharmacy, University College London, London, United Kingdom

**Keywords:** adherence, psychotropic, mHealth, smartphone, compliance, health education, mental health, digital health

## Abstract

**Background:** Patients prescribed with medication that treats mental health conditions benefit the most compared to those prescribed with other types of medication. However, they are also the most difficult to adhere. The development of mobile health (mHealth) applications (“apps”) to help patients monitor their adherence is fast growing but with limited evidence on their efficacy. There is no evidence on the content of these apps for patients taking psychotropic medication. The aim of this study is to identify and evaluate the aims and functioning of available apps that are aiming to help and educate patients to adhere to medication that treats mental health conditions.

**Method:** Three platform descriptions (Apple, Google, and Microsoft) were searched between October 2015 and February 2016. Included apps need to focus on adherence to medication that treats mental health conditions and use at least a reinforcement strategy. Descriptive information was extracted and apps evaluated on a number of assessment criteria using content analysis.

**Results:** Sixteen apps were identified. All apps included self-monitoring properties like reminders and psycho-educational properties like mood logs. It was unclear how the latter were used or how adherence was measured. Major barriers to medication adherence like patients' illness and medication beliefs and attitudes were not considered nor where information to patients about mediation side effects. Very few apps were tailored and none was developed based on established theories explaining the processes for successful medication adherence like cognitions and beliefs. Reported information on app development and validation was poor.

**Discussion:** A variety of apps with different properties that tackle both intentional and unintentional non-adherence from a different perspective are identified. An evidence-based approach and co-creation with patients is needed. This will ensure that the apps increase the possibility to impact on non-adherence. Theories like social cognition models can be useful in ensuring that patients' education, motivation, skills, beliefs, and type of adherence are taken into consideration when developing the apps. Findings from this study can help clinicians and patients make informed choices and pursue policy-makers to integrate evidence when developing future apps. Quality-assurance tools are needed to ensure the apps are systematically evaluated.

## Introduction

### Adherence to medication that treats mental health conditions

Medication adherence is usually defined as the extent that the medication taken reflects prescribed intention (Sanchez et al., 2005). Patients taking medication that treats mental health conditions have the greatest benefits when following the medication regimen compared to those prescribed with other types of medication (Zygmunt et al., 2002; Nose et al., 2003). At the same time, they are also usually difficult to adhere. For example, patients diagnosed with psychosis or depression adhere less to their medication compared to patients with physical illness (Cramer and Rosenheck, 1998).

The problem of non-adherence to medication that treats mental health conditions varies in prevalence, but is serious. Primary or continuous non-adherence ranges from 35 to 45% for patients with bipolar disorder (Colom et al., 2000; Rosa et al., 2007; Rakofsky et al., 2011), 50–60% for patients with schizophrenia (Lacro et al., 2002; Perkins, 2002), and 45% for patients with Post-Traumatic Stress Disorder (PTSD) (Rakofsky et al., 2011). The consequences of non-adherence to medication that treats mental health conditions can also be severe. For example, patients may experience increased risk of episode relapse, hospital readmission, and suicide (Angst et al., 2005; Berk et al., 2010; Murru et al., 2012). Non-adherence may also affect termination of treatment (Kemp et al., 2009) and recovery (Velligan et al., 2009). Patients may discontinue their medication and evidence are needed related to effective strategies aiming to educate and support patients with self-monitoring (Salomon and Hamilton, 2013).

The World Health Organization (WHO) published a guide for clinicians and policy makers, advising them to develop strategies in order to improve medication adherence (Sabaté, 2003). Currently in the US, the national hospital system allows clinicians to encourage their patients to use mobile health (mHealth) apps (Dayer et al., 2013). In the UK, the National Health System (NHS) introduced the Health App Library in 2013, to peer-review and list clinically safe and tailored apps for patients living in the UK. The UK national guidelines suggest tailoring support related to adherence to address the motivational and capability barriers of individuals. This approach aims to involve patients in decision making (National Institute for Health Clinical, Excellence, 2009). However, most successful interventions are usually expensive and complex (Burnier, 2000; Haynes et al., 2002; Murri et al., 2004) and mHealth apps can overcome this problem.

It is now established that patients with mental health conditions are equally likely to use mobile devices with the general population (Varshney and Vetter, 2012). The mHealth market is large with 158,000 available health-related apps (Research2guidance, 2016). These apps can be cost-effective and individually tailored to patients (Free et al., 2013). For example, combining new technologies to help patients with chronic conditions to adhere to medication has multiple benefits (Weingarten et al., 2002; Ofman et al., 2004). Despite the availability of mHealth apps, empirical analyses of their usability and benefits are lacking (Dayer et al., 2013). A recent study identified 229 available general medication adherence-related apps (Stawarz et al., 2014) but no similar evaluation is available for medication that treats mental health conditions. Also, even though using theoretical approaches can impact non-adherence (Horne and Weinman, 1999), it is not clear how these are operationalized in available apps.

### mHealth apps and unintentional non-adherence

Medication non-adherence can be unintentional (forgetting) with interventions focusing on shared goals as well as intentional (not wanting) with interventions focusing on relational strategies related to patients' goals, needs, and barriers. Interventions may have modest but significant impact and reinforcement strategies like reminders are recommended (Conn et al., 2015). The most effective, practical, and promising approaches targeting non-adherence to medication that treats mental health conditions come from combining psycho-educational interventions together with patient reminders (Mundt et al., 2001; Haynes et al., 2002; Pekkala and Merinder, 2002; Ran et al., 2003; Murri et al., 2004). Reminders usually do not involve providing the patient with information on their adherence (Dayer et al., 2013).

Using mHealth apps as reminders may also create a synergy between the patient and their clinician on decision-making. Nevertheless, very little evidence is available on how these apps support adherence using frameworks for evaluating the efficacy and effectiveness of complex interventions (Campbell et al., 2000; Craig et al., 2008) as well as evidence from non-mHealth complex adherence interventions (Nieuwlaat et al., 2014; Farmer et al., 2016).

### mHealth apps and intentional non-adherence

Adhering to medication is a health behavior which relies on decisions based on cognitive processing (Lehane and McCarthy, 2007). Social cognition models like the Common Sense Model (Leventhal et al., 1992), Social Cognitive Theory (Bandura, 1986), the Theory of Planned Behavior (Ajzen, 1991), and the Health Belief Model (Rosenstock et al., 1988) can be used to explain cognitions related to medication non-adherence. These models pertain the assumption that beliefs about illness and medication influence how patients process information and experiences (Weinman and Petrie, 1997).

Higher intentional adherence is related to greater belief on the benefits of the treatment and adequate understanding of the illness and medication (Okuno et al., 2001; Lacro et al., 2002; Perkins, 2002; Kao and Liu, 2010; Levin et al., 2015). Improving these perceptions can also improve adherence through better doctor-patient communication (Okuno et al., 2001; Lacro et al., 2002). Leventhal's Common Sense Model suggests that patients will adhere if prescriptions make sense in light of their beliefs about medication and their past experiences (Leventhal et al., 1992). The Capability, Opportunity, and Motivation (COM-B) model suggests that health behaviors are influenced by how motivated and capable is an individual (Michie et al., 2011). Moreover, the Necessity-Concern model refers to how patients compromise their concerns against their perceived necessity to take their medication and has clinical benefit on explaining non-adherence (Horne and Weinman, 1999). One study suggests that this model may explain non-adherence more than any other risk factor (Kalichman et al., 2015). These models constitute useful frameworks when evaluating the effectiveness of interventions aiming at changing intentional non-adherence.

Smartphone apps usually target barriers to medication adherence, which are complex and interactive. Most of the barriers are patient-controlled and therefore, indicative of the patient's important role on following the clinicians' prescriptions (Osterberg and Blaschke, 2005). Patients' motivations and experiences are particularly relevant for medication that treats mental health conditions (Swarbrick and Roe, 2011). As a result, an individual's perceptions about medication and their illness can influence their intentional non-adherence. The use of mHealth apps for medication adherence comes with benefits for the patient (ease of access, mobility, opportunity for tailoring features) and potentially for the health system (cost-effectiveness by less use of the system's facilities).

### This review

Evidence of theoretical or empirical frameworks informing the medication adherence apps' properties is needed to allow for comparison between the apps. At the same time rigorous evaluation will determine their efficacy. Previous research is focused on general adherence apps (Dayer et al., 2013) whilst this study evaluates the apps' aims and functioning. The aim of this study is to review all mHealth apps available in 2016 targeting intentional non-adherence to medication that treats mental health conditions.

## Methods

### Eligibility criteria

Smartphone apps focused on medication that treats mental health conditions for use by the general public were included in the search. The apps had to include multiple reinforcement strategies that target intentional non-adherence (i.e., reminder systems which target unintentional non-adherence only, with no other strategy included were not selected). Reinforcement strategies include properties like logs, reminders, and psycho-education. Finally, the search was restricted in apps that included a description in English in the web platforms irrespective of the apps' language functioning.

### Search strategy and app selection

Three mobile platforms were screened, Apple App Store for iPhone, Google Play for Android and Microsoft Store for Microsoft smartphones. These platforms were searched since Apple and Google include the 90.6% of available medication adherence apps internationally (Dayer et al., 2013) whilst there is no previous evidence for available apps for Microsoft smartphones. Moreover, Apple and Google are the two most popular platforms that account for 90% of smartphone users (Malhotra, 2014). The following search keywords were used: “medication adherence,” “medication monitoring,” “medication compliance,” “dose,” “drug,” “med(s),” “medication(s),” “reminder,” “intake,” and “treatment.” All data were collected in October 2015 and an update followed in February 2016. The chosen period of time was pragmatic and reflects the availability in that specific period but the learning outcomes help with developing suggestions for future apps' development. It also follows from a previous review of generic adherence apps (Dayer et al., 2013). Three authors searched the platforms and screened the app descriptions independently and all apps that met the inclusion criteria were included. A fourth author crosschecked the apps to confirm eligibility. The most common reasons for excluding apps was: no focus on mental health and/or medication that treats mental health conditions (general medication apps), apps focusing on information provision only, no English description available in the platforms and apps for entertainment.

### Data extraction and analysis

The descriptions that were available in the platforms through the searches were assessed and data extracted using two types of information: descriptive information and assessment criteria. Based on both types of information, an assessment form was developed for data extraction. The descriptive information assessed were: developer, developer's background (company, individual, or charity/non-governmental organization), country of origin, cost (free or not), platforms the app is available, targeted mental health condition(s), the latest update reported and descriptive information available in the online platforms.

The evidence from the app descriptions in the platforms were synthesized and evaluated based on a list of pre-defined criteria. The assessment criteria considered (Table 1) were derived from a scoping review of the literature related to using mHealth apps for medication adherence (Sackett and Haynes, 1976; Haynes and Sackett, 1979; Horne, 1998; Horne and Weinman, 1998; Osterberg and Blaschke, 2005), the NHS NICE guidelines for medication adherence (National Institute for Health and Clinical Excellence, 2009) and adherence-related theoretical frameworks like the COM-B, the Common Sense and the Necessity-Concern models. The assessment criteria were also evaluated in terms of their relevance and usefulness by one author (RH) with significant experience in medication adherence research. They were grouped into six groups: patient education/content, psycho-education/strategy, patient self-monitoring, personalization, interactivity, and “other.” The criteria are defined and operationalized in Table 1. Then, the online descriptions were searched for any reference to any peer-reviewed papers in order to determine the evidence-base for the apps' development, design and evaluation. Additionally, the apps' names were used as keywords in two popular health-related search engines, PubMed and PsycInfo.

**Table 1:** The assessment criteria for properties of smartphone applications for adherence to medication that treats mental health conditions.

**A/A**	**Name**	**Definition**
**PATIENT EDUCATION/CONTENT**		
A	Information about medicine and its side effects	Information in the app on prescribed medication characteristics and side effects
B	Information about alternative treatments	Information in the app on any alternative treatments (i.e. relaxation techniques)
C	Information about clinicians and/or pharmacists near the user	Information in the app that can be used by the user to access local clinicians and/or pharmacists
**PSYCHO-EDUCATION/STRATEGY**		
D	Use of psycho-educational approaches	Use of psycho-educational techniques, which aim to empower patients to control their health (i.e. motivation, monitor health-state, rewarding etc.)
**PATIENT SELF-MONITORING**		
E	Medication logs	Logs that allow the patient to record when the patient takes prescribed dose
F	Other logs	Other logs which allow patient to record their mood, health history etc.
G	Medication reminders	Reminders for patient to take prescribed medication
H	Appointment reminders	Reminders for patient to attend an appointment with a health professional
I	Dose tracking (missed and taken)	Logs that allow the patient to record the dose they take
**PERSONALIZATION**		
J	Tailoring properties	Use of tailoring techniques to allow the app to be customized to patients' needs (i.e. in terms of patients' beliefs, perceptions, gender etc.)
**INTERACTIVITY**		
K	Medication logs available to clinicians	Logs of patients' records of taken medication available to their clinician
L	Interaction properties with clinicians/hospitals etc.	Use of interactivity properties allowing the patient to communicate with health professionals
**OTHER**		
M	Availability in multiple platforms	App available in more than one platform
N	Availability in other languages	App available in more than one language

Three authors performed all data extraction. Any discrepancies were resolved by consensus within group discussions with a fourth author. To increase reliability of assessment the 20% of assessment forms for each author was double-checked by a second author performing an independent assessment. Content analysis and critical appraisal were then performed on information available on the online descriptions of the identified apps. All emerging findings were discussed between the authors whose diverse experience and expertise facilitated the discussion of findings.

## Results

### Descriptive information

Using combinations of the keywords, eighty apps were identified as potentially eligible after removing duplicates. These were the apps that were identified using the keywords before screening based on the inclusion criteria. Sixteen unique smartphone apps were identified that met the eligibility criteria. The app characteristics are summarized in Table 2. Six apps were developed in the USA, two in Spain whereas one each was developed in Australia, Brazil, Canada, China, Egypt, India, Ireland, and the UK. The majority of apps were commercial and developed by companies (11, 68.75%), four were developed by charities or non-governmental organizations (25%) and one developed by an un-specified group of individuals (6.25%). Universities or other academic or research institutions developed none of the available apps. Half of the included apps were available for both Apple iPhone and Google Android devices (8, 50%), a third available for Google Android devices only (5, 31.25%), two available for Apple iOS, Google Android, and Windows devices (12.5%) and one for Apple iOS devices only (6.25%). Overall, the majority of apps were available to download for free (13, 81, 25%), with the remaining requiring a fee.

**Table 2:** Smartphone applications for adherence to medication that treats mental health conditions.

**A/A**	**Name**	**Owner/programmer**	**Country**	**Cost**	**Platform(s)**	**Condition(s)**	**Latest update**	**Description**
1	Intervention911	Ken Seeley Communities (Intervention 911)^a^	USA	Free	And/App	Addiction or mental illness	11/06/14 for And 19/06/14 for App	Intervention911 is designed for patients suffering from any type of mental disorder or addiction. The app helps the users to outline their recovery plan, set up reminders, track medication, “check-in” to therapy appointments or find a location for drug testing. It also provides data for High Licensed Professional Boards, Treating Doctors, Treating Therapist, Drug Testing Company, Medication Compliance etc. The data are strictly confidential. Further, it is possible for the users to have proof of their recovery progress through a generated report.
2	TARA: Mental Health Nurse (And) TARA: Mood Log and Medication Reminder (App)	(Patient@Center)^b^	USA	With fee	And/App	All mental health conditions (Tracks Mood, anxiety or sleep)	09/09/14 for And 15/09/14 for App	Both the general public and mental health professionals can use this app. The app helps the user adhere to their medication through reminder and by using a Mood Log to track mood, anxiety, sleep and stressors. When the medications make the user “feel better” they can see the impact on a graph. They can also get points for checking in with the app and taking their medications daily. A daily cliffhanger quiz question is used to motivate users to use the app. If their mood, anxiety or sleep becomes worse than “standard thresholds,” the app provides a warning. The user can then enter their detailed symptoms and request an appointment with their mental health professional. The app enables a mental health professional to view summarized information for all their patients.
3	My Mind Western Trust	Troll inc/Tom Brewster (in App)^a^	Ireland	Free	And/App/Mic	All mental health conditions	26/08/14 for And 11/06/2014 for App	The app supports people with mental health conditions. It provides information concerning mental health conditions as well as the types of treatment and medication. Users can set reminders for medications and health appointments. It includes a map of mental health recovery services in the Western Trust area in Northern Ireland.
4	LIFE:)	High Definition Services Natasha Staunton (in App)^a^	Australia	With fee	And/App	ADHD, ADD, Asperger's, Depression and physical chronic illness	18/08/15 for And 01/09/2015 for App	The app concerns both mental and chronic physical illness (chronic illness) management, such as, ADHD, Asperger's Syndrome, depression, diabetes, and blood disorders etc. Users can manage their pill reminders, triggers, food and mood diaries and view and email reports to health professionals.
5	Life Reboot - Fight Depression	Photonapps^a^	India	Free	And	Depression	28/10/15	This app concerns depression and is designed as a support to the treatment. It contains six modules: anonymous forum, diary, medicine reminder, daily motivational quotes, jokes and games (Tic-tac-toe, painting).
6	Medica Reminders	IRWAA LLC^a^	Egypt	With fee for And/ Free for App	And/App/Mic	ADHD/ADD and other	03/01/16 for And 24/06/14 for App	The app helps patients and caregivers manage prescriptions and take/give the right medications on the right time.
7	ADDA Health Storylines	Health Storylines^c^	USA	Free	And/App	ADHD	31/08/15 for And 27/01/16 for App	The app aims to motivate adults with ADHD by encouraging habit building and helping to track day-to-day activities.
8	KnKt'd Behavioral Health	Synergistic Creations, LLC^a^	USA	Free	And	All mental health conditions	31/08/15	The app includes an electronic online system for behavioral health purposes featuring technology that allows the users to collaboratively upload information that is tracked and stored daily in a database. The information is then configured into graphs and charts for later review by one or more users
9	Booster Buddy	Island Health^c^	Canada	Free	And/App	All mental health conditions	18/11/15 for And 20/11/15 for App	The app suggests that it is designed to help teens and young adults “improve their mental health.” It allows users to earn achievements as a “sidekick” guides them through a series of daily quests designed to establish and sustain positive habits.
10	Mood Tracker By: CTHF^§^	Cheryl T. Herman Foundation^c^	USA	Free	And/App	Depression, Bipolar disorder		The app aims to help individuals with depression or bipolar disorder “manage their health.” It provides an easily understandable picture that users and their doctors can evaluate together, review and change treatment plans where necessary, and provide a way to monitor the course of treatment response through daily charting.
11	Your Medicine 1-2-3 pro	Sombrero Mobile^a^	Brazil	With fee	And	All mental health conditions (special property for ADD)	11/12/13	The app allows users having full support for time zones. There is support for take-as-needed medications, support for contraceptives, and support for when users have to keep strict “interval-based control.”
12	uMotif	uMotif Digital Health^a^	UK	Free	And/App	Parkinson's disease, other physical illness	29/06/16 for And 01/02/16 for Apple	The app helps users to track data relevant to their health, set up medication and task reminders and engage with content selected by their clinician. The data they enter is used to create their individual Health Report.
13	Start - medication manager for depression: pill reminder, mood & side effect tracker	Iodine Inc.^a^	USA	Free	App	Depression	02/02/16	The app aims to help users decide if the medication is working as expected for their depression. It allows users to track their side effects, monitor their mood, set goals, learn from others' experience, and decide how well their antidepressant is working. Every 2 weeks, the app provides reports with options to discuss with their health professional.
14	Mental Wellness Everyday	HKAPR.ORG^c^	China	Free	And/App	Schizophrenia	14/06/14 for And 20/06/14 for App	The app aims for patients with schizophrenia, family members or health professionals (including case managers, doctors and nurses). The app includes medication reminder, drug adherence recording, mood tracking and personal goals setup. It also contains encouraging quotes and award virtual badges to the patients that achieve high drug adherence and participation of personal goals.
15	ADHD Adults	Labs Health Company^a^	Spain	Free	And	ADHD	07/01/16	This app aims to help adults with ADHD track and monitor their medical treatment. Users can also built their medical history, plan their daily activities, measure treatment results and use self-assessment questionnaires. Users can have a direct communication with their doctor through the ADHD Doctors app.
16	ADHD Kids	Labs Health Company^a^	Spain	Free	And	ADHD	07/01/16	The app aims to assist parents, caregivers and teachers for the coordination of the follow up of children under 18 with ADHD. Users can manage medical treatments, plan everyday activities, review treatment results, use auto-evaluation tests, as well as weight and height integrated percentile calculator. Also, this app allows the real time communication of several tutors in a collaborative way.

*And, Android; App, Apple; Mic, Microsoft; ADHD, Attention Deficit Hyperactivity Disorder; ADD, Attention Deficit Disorder*.

a *Commercial*,

b *Individual(s)*,

c Charity or Non-Governmental Organization (NGO).

§ *Available at: http://apk4ios.com/APK_Mood-Tracker-By-CTHF_iOS.html*.

Among the apps, the range of update dates was between 2013 and 2016. Overall, one was updated in 2013, 5 in 2014, 5 in 2015 and 6 in 2016. Two apps had their iOS and Android versions update in different years (Medica reminders and ADDA Health Storylines). However, updates can be anything from scientific update to a technical (bug) update.

### Properties based on assessment criteria

All of the available apps included more than one type of properties for medication adherence (Table 3). For example, Life Reboot—Fight Depression included only patient self-monitoring properties (i.e., medication reminders), while ADDA Health Storylines included all properties from the pre-specified assessment criteria except patient education/content. The patient self-monitoring group of criteria, which includes properties related with the ability of the patients to monitor their daily-prescribed medication use, was met in all 16 apps. Of the patient self-monitoring criteria, all apps used medication reminders (criterion G), and 14 included medication logs (criterion E, 87.5%). On the other hand, tailoring-related properties were found only in four applications (25%), representing the less applicable criterion. A third of the apps were developed in order to address all mental health conditions (6, 37.5%) or ADHD and/or ADD (5, 31.25%). In addition, all apps except one (My Mind Western Trust) included also information about other log properties (criterion F, 93.75%). Mood log and agenda/diary were the most frequently used extra logs (Table 4). Finally, only 2 apps (12.5%) were available in other languages than English (ADHD Adults and ADHD Kids).

**Table 3:** Properties of smartphone applications for adherence to medication that treats mental health conditions.

**A/A**	**Name**	**Assessment criteria**													
		**Information about medicine and its side effects**	**Information about alternative treatments**	**Information about clinicians and/or pharmacies near the user**	**Use of Psycho-educational approaches**	**Medication logs**	**Other logs (see Table 4 for details)**	**Medication reminders**	**Appointment reminders**	**Dose tracking**	**Tailoring features**	**Medication logs available to clinicians**	**Interaction features**	**Availability in multiple platforms**	**Availability in other languages**
1	Intervention911			♦^a^		♦	♦	♦	♦	♦		♦^b^		♦	
2	TARA: Mental Health Nurse				♦^c^	♦	♦	♦		♦		♦	♦^d^	♦	
3	My Mind Western Trust	♦	♦	♦^e^				♦	♦					♦	
4	LIFE:)					♦	♦	♦				♦^f^		♦	
5	Life Reboot - Fight Depression						♦	♦							
6	Medica Reminders				♦^g^	♦	♦	♦	♦	♦		♦		♦	
7	ADDA Health Storylines				♦^h^	♦	♦	♦	♦		♦^i^		♦	♦	
8	KnKt'd Behavioral Health					♦	♦	♦	♦		♦	♦	♦		
9	Booster Buddy		♦^j^		♦^k^	♦	♦	♦	♦	♦				♦	
10	Mood Tracker By: CTHF		♦^l^			♦	♦	♦		♦		♦	♦^m^	♦	
11	Your Medicine 1-2-3 pro					♦	♦	♦		♦	♦	♦^n^		♦	
12	uMotif					♦	♦	♦	♦	♦	♦	♦^o^	♦^p^	♦	
13	Start - medication manager for depression	♦			♦^q^	♦	♦	♦							
14	Mental wellness everyday		♦^r^		♦^s^	♦	♦	♦	♦	♦				♦	
15	ADHD Adults				♦^t^	♦	♦	♦		♦		♦	♦		♦^u^
16	ADHD Kids				♦^t^	♦	♦	♦		♦	♦^v^	♦	♦		♦^u^
Total N (%)	2/16 (12.5)	4/16 (25)	2/16 (12.5)	8/16 (50)	14/16 (87.8)	15/16 (93.8)	16/16 (100)	8/16 (50)	10/16 (62.3)	5/16 (31.3)	10/16 (62.3)	7/16 (43.8)	11/16 (68.8)	2/16 (12.5)	

a *Local support services*,

b *Ability to get report about recovery process*,

c Rewarding points, graphs, quiz questions, worsening warnings, mood graph, daily quiz, points for daily check in, notification if mood/anxiety/sleep threshold worsens

d *Request for appointment with the clinician, ability for the clinicians to pull up the information of the patient through their app*,

e *Western trust area*,

f *Ability to email reports to health professionals*,

g *Feature for audio-recording clinician's medical instructions*,

h *Motivation features to complete typically mundane tasks by documenting achievements*,

i Record weights and other “vitals,”

j Self-care routines and “real-life socialization,”

k *Rewarding and worsening points*,

l *Relaxation and stress-relief meditation techniques*,

m *Email option for exporting graphs and reports to share with clinician*,

n *The accumulated information can be emailed to your doctor*,

o *Caregiver provides password for the patient and the patient can show a health report*,

p *Not immediate*,

q *Helpful tips from pharmacists and expert patients, progress reports for monitoring purposes*,

r *Comparison of different types of antipsychotics on efficacy, durability and compliance with dosage*,

s *“Knowing about schizophrenia” section, targets for self-improvement, badges*,

t *Treatment results measurement, status analysis using self-assessment questionnaires, medication related charts*,

u *Spanish*,

v *Height and weight monitoring graph*.

**Table 4:** Information about other log properties except medication logs available in smartphone applications for adherence to medication that treats mental health conditions.

**Name**	**Other logs**
1. Intervention911	Record and count sober days
2. TARA: Mental Health Nurse	Mood log (anxiety, sleep, stressors), health history
3. My Mind Western Trust	None
4. LIFE:)	Triggers, mood and food diaries
5. Life Reboot - Fight Depression	Diary
6. Medica Reminders	Doctor follow-up
7. ADDA Health Storylines	Document patient health and daily living with a journal
8. KnKt'd Behavioral Health	Feel, utilization of coping skills and use of your supports
9. Booster Buddy	Mood log
10. Mood Tracker By: CTHF	Mood log
11. Your Medicine 1-2-3 pro	Emergencies, “about me,” medicines, doctors, pharmacies, hospitals, health plans and extra info.
12. uMotif	Health report, data tracking, tasks list, daily diary, content selected by the clinician
13. Start - medication manager for depression	PHQ-9 survey and check in every few days with the PHQ-2 on their mood and medication.
14. Mental Wellness Everyday	Targets and tips for self-improvement, mood log (“emotional index”), obsessions, contact resources
15. ADHD Adults	Interactive agenda for daily life activities, medical history
16. ADHD Kids	Interactive agenda for daily life activities, medical history, height and weight percentile

### Use of psychological parameters

**Figure 1:**
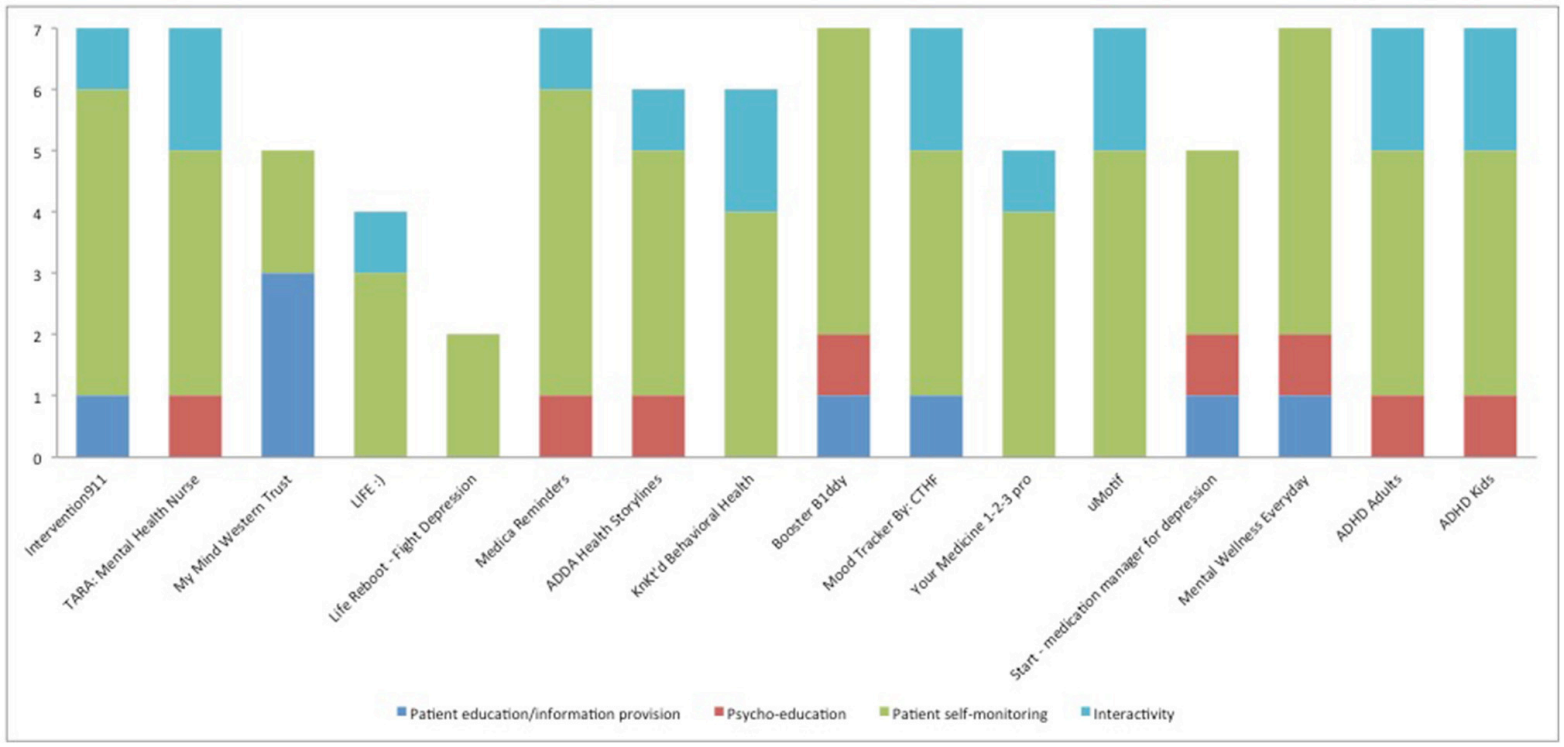
Characteristics of available apps for adherence to medication that treats mental health conditions based on number of properties under each assessment criteria. The Y axis consists of number of properties related to each group of assessment criteria (outlined in detail in Table 3).

In general the available apps were focused on self-monitoring and much less on psycho-education/strategy (Figure 1). Among the psycho-educational properties, mood logs were used in six apps (37.5%) but it is not clear how mood was used except in one app. The TARA app uses mood, anxiety, sleep, and stressors tracking to demonstrate graphically to the patient the positive impact of adherence (when the patient “feels better”). The mood logs were mostly used as a wider self-management technique and to warn the patient when they report lower levels of mood than the “standard thresholds.” No information is available on these thresholds. In the rest of the apps, like Start-Medication Manager for Depression, the mood logging aims to enable the patient to self-evaluate whether the app is improving their condition or not. Surprisingly there were interactivity properties in a number of apps but it was the patient who could share information with their health care providers rather than using an interactive channel with providers to enable communication.

### Apps' development and evaluation

None of the available apps acknowledge or reference any peer-review study or any empirical-based method of development. Moreover, the input of health professionals is unclear even though in some cases they were involved in the development of the apps. In addition, when the search engines were searched (PubMed, Medline, PsycInfo) none of the apps were found to be validated or being in progress of validation. Finally, only one app reported using a validated screening measure (Start-Medication Manager for Depression) but none reported using a validated method of assessing medication adherence.

## Discussion

This study provides evidence about 16 smartphone apps across three different platforms aiming to educate or help patients adhere to medication that treats mental health conditions. Similarly to other types of medication adherence interventions, the smartphone apps focus on reminders, reinforcing techniques, psycho-education, providing information and counseling (Haynes et al., 1996; Graves et al., 2010). It is promising that included apps are not used only as reminders but combine a range of techniques. On the other hand, illness and medication beliefs are not taken into consideration and apps' development is not theory-based. Measuring adherence is one of the challenges in the field since no gold standard method exists. However, none of the available apps report a validated method of measuring adherence. A range of self-report measures exist with satisfactory reliability and validity which constitute possible options (Garfield et al., 2011). For example, a version of the Medication Adherence Report Scale (Horne and Weinman, 1999, 2002) or a Visual Analogue Scale can be used to assess whether the patient adheres to medication. Using medication logs can be useful in terms of providing information about the user's daily intake, but evidence suggests that combining two methods (in this case validated questionnaires and medication logs) can lead to a more valid assessment of patients' adherence (Barnestein-Fonseca et al., 2011). It is important to highlight that medication adherence is a relational issue and mHealth apps if validated can contribute toward shared decision-making. In this context, patients' goals and targets are explored with the clinician and the patient is able to monitor outcomes in a continuous learning process.

The COM-B model (Michie et al., 2011) can be used as useful framework when apps target patients' perceptions about medication and illness, their motivations or their ability to adhere (i.e., reminders). Collecting information about the patients' beliefs about illness and medication can help tailoring the app, which is the less common criterion. Tailoring can potentially improve both the sustainability of using the apps but also non-adherence, if the apps help with changing perceptions (Williams et al., 2008; Gatwood et al., 2014). Poor tailoring was also recently reported in a recent evaluation of health behavior change apps (McMillan et al., 2016).

The level that patients' emotions are taken into consideration in the included apps is not clear. For example, none of the apps makes use of patients' concerns toward medication. A third of the apps use mood logs for self-management but it is not clear how mood is used to improve non-adherence except in one (TARA: Mental Health Nurse for Android and TARA: Mood Log and Medication Reminder for iOS). An app may be more acceptable by patients if it takes into account how they feel and when. It is also surprising that information about coping with side effects is included only in two apps. Side effects can be a serious barrier to medication adherence and patients can benefit if they have information about their medication's side effects. Assessment of emotions, depression and anxiety is insufficiently used. App developers and researchers should consider using the Experience Sampling Method (Csikszentmihalyi and Larson, 2014) to reduce bias of assessment. Apps like PsyMate can be used as a point of reference to overcome validity and reliability biases of assessment of moods (Boyce, 2011).

Furthermore, none of the apps takes into account patients' perceptions about the necessity to take their medication even though this is one of the strongest predictors of intentional non-adherence (Horne and Weinman, 2002; Horne et al., 2004; Clifford et al., 2008). The Necessity-Concern Framework (Horne and Weinman, 1999) can be used in order to assess and modify patients' concerns of prescribed medication and how necessary they perceive them to be. If patients' perceptions are assessed the intervention can be targeted if the patient has negative perceptions that may compromise adherence. Successful ways of influencing these perceptions can be the focus of future research.

Even though the focus of this review is intentional non-adherence, there are properties that target unintentional non-adherence like reminders. Medication reminders are the most popular properties in the included apps. On the other hand, none of the apps is a medication reminder only—they combine reminders with other reinforcement techniques. This holistic approach designates both process-based and relational approaches next to reminders that target unintentional non-adherence. Therefore, there are various ways medication reminders can work to tackle prospective and retrospective memory which both influence adherence (Kliegel et al., 2008). Future applications may consider tailoring reminders and using theory-driven concepts and algorithms to determine timing of messages which was recently proposed (Gatwood et al., 2014). However, non-adherence may not be a one-dimensional concept; it can be intentional (consciously deciding) or unintentional (forgetting). Recent evidence suggest using both when designing interventions in clinical trials (Wroe, 2002; Atkins and Fallowfield, 2006; Clifford et al., 2008; Iihara et al., 2014). Even if it is positive that a number of apps use a variety of properties that stem from both types of adherence, very few apps assess the patients' type of non-adherence. Therefore, it is difficult to tailor the properties to whether the patient intentionally or unintentionally does not adhere to medication. For example, pill reminders can be helpful when non-adherence in unintentional but not when it is intentional. The efficacy of the apps can be compromised if reminders are provided to patients who intentionally do not adhere to their medication. Reminders may tackle unintentional adherence whilst psycho-educational properties may tackle intentional adherence.

Interventions using tailored apps combining psycho-educational properties with patient self-monitoring and reminders have the greatest potential to improve adherence (Williams et al., 2008). Moreover, educating patients can be one of the most effective approaches in medication adherence (Haynes et al., 2008; Conn et al., 2009). Psycho-education/strategy is used in half of the available apps but it is not clear what the content and validity of the psycho-education/strategy properties are for each app.

Furthermore, interactivity with health professionals and the patients' family are used in ten of the identified apps but with limited information on how this is implemented. For example, patients can email adherence reports to health professionals or share the report during the consultation. For the ADDA Storylines app there is also the opportunity to provide information immediately to the family of the patient. Interactivity has the potential to keep the health professionals and family informed if the patient is non-adherent in a peer-support model of patient education. More research is needed on how to safely and accurately share patients' information or help with referrals when psychological interventions may be needed or shared decision-making.

Addressing mental health conditions can be implemented more successfully using evidence-based treatments and properties. There was a call recently for Universities to adopt a more active role in the research and development of mHealth apps (Kumar et al., 2015). However, no academic institute or professional organization other than commercial companies and NGOs/Charities were involved in the development of the identified apps. This study confirms recent evidence that empirical analyses of the efficacy of medication adherence apps is lacking (Sposaro and Tyson, 2009; Wohlers et al., 2009; Dayer et al., 2013). Based on this, there is a need for studies to test the validity, utility, and efficacy of the available apps. In relation to implementation of apps into healthcare, a recent report suggested an Evidentiary Standards Model to ensure patient safety there is a need for at least two efficacy and two effectiveness trials and evidence on dissemination and cost-effectiveness (Tomlinson et al., 2013). On the other hand, it is possible that evidence was taken into consideration during the development of the apps but this is not clearly stated in the app descriptions and no peer-reviewed paper is available on apps' development.

There is also a need to publish the development process of the apps so that evaluation and validation is possible. Research and evaluation should take place during the development process, with input from health professionals, patients and application developers in order to improve the apps' feasibility and usability. “Co-creation” with patients during the development stage is necessary so that apps reflect patients' needs and so that acceptability is improved. This was demonstrated in a recent study with patients with schizophrenia, which revealed that usability testing led to overcoming system vulnerabilities (Ben-Zeev et al., 2013). It was also highlighted in a review of apps for health behavior change where evidence on collaboration with health professionals or patients and apps' evaluation was also lacking (McMillan et al., 2016). Even though studies on the efficacy of available mHealth apps for medication adherence are limited, the evidence demonstrates the potential for benefits for the patients if well designed and validated. It is important to note that it is positive that apps exist with different and diverse properties. Not all apps should have the same functioning but differently designed apps may work in different aspects of medication non-adherence.

### Limitations

To our knowledge, this is the first study that evaluates the content of apps related with adherence medication that treats mental health conditions but limitations should be taken into consideration. The study captures the market space at a given time (late 2015, early 2016). As a result, it provides the state of knowledge and availability of properties on that given time period because the three most popular web platforms with a large user base were reviewed. However, we acknowledge that this is a fast growing area and different apps might emerge. On the other hand, learning points from this study can inform future apps' development. The study is also limited by the fact that even though some of the apps were downloaded for testing the analysis is based on the app descriptions. However, users should be made aware of the app development process and the evidence taken into consideration before they download an app so they can make an informed choice. Only three platforms were reviewed and other smaller platforms may have other apps but more likely these small platforms will use the same apps with the ones reviewed. The study is restricted to apps with English language used in the web platforms. However, since the majority is developed in the USA, it is unlikely that a lot of apps were missed. In this study PubMed was used to search for papers referring the apps but papers in other databases may have been missed or papers referring to the apps in different names. However, the focus of the evaluation was on whether there is available evidence to ensure patient safety similarly to the Evidentiary Standards Model (Tomlinson et al., 2013). Finally, we may have missed some generic apps because of the restrictions created by the inclusion criteria. However, even if some apps are missed we are confident that the current review captures the majority that aim to help patients to adhere to medication and provides insights into how these are developed.

### Practice implications

The area of mHealth can contribute toward personalized medicine. A more holistic approach which takes into consideration both intentional and unintentional non-adherence is useful to design effective mHealth apps. Strategies that target intentional non-adherence can also be useful in terms of medical tapering which refers to gradual discontinuation or reduction of medication. Medication tapering can be benefited by approaches that lead to shared-decision making. There is a need for scientifically and empirically validated apps combining research, clinical experience, and marketing policy. Moreover, systemic evaluation of existing apps in real-life conditions is necessary to provide evidence on how apps can be used within the current health system. Developers are encouraged to use an evidence-based approach in designing and developing similar apps. This study may inform mental health professionals who are encouraging the use of apps as well as the patients that use them to make an informed choice. The current study highlights the need for developing quality assurance tools for medication adherence apps. It can inform policy-makers on the need of integrating evidence-based knowledge into app development and evaluation and pushing for regulating and monitoring the large and fast growing mHealth field. Finally, more time should be spent in co-creating the apps with patients. If apps are validated, this may potentially influence clinicians' confidence in recommending them and policy makers into integrating these interventions into the local health systems.

Future apps that target intentional non-adherence need to improve patients' understanding of mental health conditions and medication attitudes (Kao and Liu, 2010; Levin et al., 2015) to address the negative attitudes toward daily medication and barriers of coping with daily routines (Sajatovic et al., 2009) and help patients setting their goals. Finally, it would be useful if apps could identify if the patient is intentionally or unintentionally non-adherent. Co-creation of apps with patients and health professionals is highlighted to increase the possibility of more efficacious, acceptable, and useful interventions (Camerini et al., 2013).

## Author contributions

AK and GG conceived the study initially with the contribution of EP and AD in study design. The initial searches were conducted by GG, EP, and AD and discussed between the three authors and AK. The assessment criteria were drafted by AK and GG with RH contributing to validation. AK drafted the first draft with all authors contributing with critical comments. All authors approved the final draft.

### Conflict of interest statement

The authors declare that the research was conducted in the absence of any commercial or financial relationships that could be construed as a potential conflict of interest. The handling Editor declared a shared affiliation, though no other collaboration with two of the authors, APK and RH.
